# Cell fusing agent virus rarely transmits vertically in artificially infected laboratory-colonized *Aedes aegypti* mosquitoes

**DOI:** 10.1186/s13071-024-06232-6

**Published:** 2024-04-04

**Authors:** Dilip K. Nag, Kathryn Efner

**Affiliations:** Arbovirus Laboratory, Wadsworth Center, New York State Department of Health, 5668 State Farm Road, Slingerlands, NY 12159 USA

**Keywords:** Arbovirus, Insect-specific virus, Orthoflavivirus, Cell fusing agent virus, Mosquitoes, Vertical transmission

## Abstract

**Background:**

Vertical transmission (VT) of arboviruses (arthropod-borne viruses) can serve as an essential link in the transmission cycle during adverse environmental conditions. The extent of VT among mosquito-borne arboviruses can vary significantly among different virus families and even among different viruses within the same genus. For example, orthobunyaviruses exhibit a higher VT rate than orthoflaviviruses and alphaviruses. Mosquitoes are also the natural hosts of a large number of insect-specific viruses (ISV) that belong to several virus families, including *Bunyaviridae*, *Flaviviridae*, and *Togaviridae*. Cell fusing agent virus (CFAV), an insect-specific orthoflavivirus, displays higher VT rates than other dual-host orthoflaviviruses, such as Zika and dengue viruses. High VT rates require establishment of stabilized infections in the germinal tissues of female vectors. To delve deeper into understanding the mechanisms governing these differences in VT rates and the establishment of stabilized infections, the ovary infection patterns and VT of Zika virus (ZIKV) and CFAV were compared.

**Methods:**

Laboratory colonized *Aedes aegypti* females were infected with either ZIKV or CFAV by intrathoracic injection. Ovary infection patterns were monitored by in situ hybridization using virus-specific probes, and VT was determined by detecting the presence of the virus among the progeny, using a reverse-transcription quantitative polymerase chain reaction (PCR) assay.

**Results:**

Both ZIKV and CFAV infect mosquito ovaries after intrathoracic injection. Infections then become widespread following a non-infectious blood meal. VT rates of ZIKV are similar to previously reported results (3.33%). CFAV, on the contrary transmits vertically very rarely. VT was not observed in the first gonotrophic cycle following intrathoracic injection, and only rarely in the second gonotrophic cycle. VT of CFAV is mosquito population independent, since similar results were obtained with *Aedes aegypti* collected from two different geographic locations.

**Conclusions:**

Although CFAV infects mosquito ovaries, the occurrence of VT remains infrequent in artificially infected *Ae. aegypti*, despite the observation of high VT rates in field-collected mosquitoes. These results suggest that infections of insect-specific viruses are stabilized in mosquitoes by some as yet unidentified mechanisms.

**Graphical Abstract:**

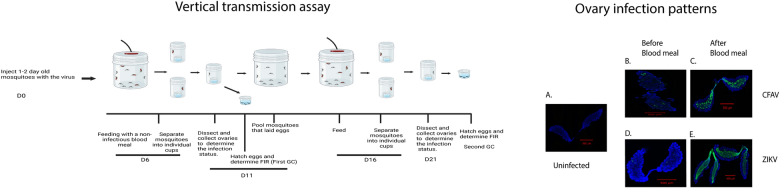

**Supplementary Information:**

The online version contains supplementary material available at 10.1186/s13071-024-06232-6.

## Background

The transmission of pathogens from one host to another occurs either by vertical or horizontal transmission [1]. Vertical transmission (VT) is defined as direct transmission of pathogens from infected parents to their offspring. Horizontal transmission (HT) encompasses all other modes of transmission. Mosquito-borne arboviruses (arthropod-borne viruses) include several clinically important arboviruses, such as dengue (DENV), Zika (ZIKV), yellow fever, West Nile (WNV), and chikungunya (CHIKV) viruses. These viruses are maintained in nature by a combination of both VT and HT. Within the mosquito population, viruses can be maintained by VT and venereal transmission during mating between an infected and an uninfected mosquito. VT is believed to play an important role in viral maintenance in nature during extreme weather conditions, such as dry seasons in tropical areas or cold seasons in temperate regions [2]. VT can also aid in preserving a virus within a particular area, especially when the concentration of the susceptible vertebrate hosts is low due to vaccination or natural infection [3].

The ability of mosquitoes to spread a pathogen is closely tied to their reproductive strategy, as the majority of mosquitoes require vertebrate blood to produce a batch of eggs. The cycle of blood feeding, egg production, and egg laying is collectively known as the gonotrophic cycle (GC). Mosquitoes generally become infected with the virus during blood feeding on a viremic vertebrate host. After replication at the initial infection site (e.g., midguts), viruses disseminate to secondary tissues such as salivary glands and ovaries, rendering the host mosquito capable of transmitting the virus to another susceptible host during subsequent blood meals or to the progeny via infected eggs.

VT has been studied in the past either by using field-collected mosquitoes or through the use of experimentally infected mosquitoes in the laboratory. These studies have shown that VT is modulated by several factors, including viral and mosquito taxa, GC, climate, and bacterial infection status of the vector [4]. For example, *Aedes* mosquitoes exhibit higher VT rates (VTRs) than *Culex* mosquitoes, and orthobunyaviruses show higher VTRs than orthoflaviviruses and alphaviruses. VTRs also depend on the GC. Offspring produced during the second or later GCs display higher VTRs than offspring produced in the first GC after an infectious blood meal, suggesting that either the virus is not disseminated to the ovaries in the first GC, and/or that the ovaries become more permeable during second and later GCs [5–7]. In addition, *Aedes* mosquitoes show higher VTRs under arid climatic conditions compared with equatorial or warm climatic conditions [4]. Finally, the bacterial infection status of the mosquito determines the VTRs. Infection of vector mosquitoes with *Wolbachia* has been demonstrated to have both positive and negative effects on VTRs [8, 9].

Additionally, mosquitoes serve as natural hosts of a wide range of insect-specific viruses (ISVs) that belong to several virus families, including *Bunyaviridae*, *Flaviviridae*, and *Togaviridae* [10]. Because of this host specificity, there are no vertebrate amplifying hosts capable of sustaining a complete viral life cycle between mosquitoes and vertebrate animals. These insect-specific viruses (ISVs) persist in nature mainly via VT and venereal transmission, and possibly through horizontal transmission during the larval stages in their aquatic habitat [10–15]. Insect-specific orthoflaviviruses (ISOFs) are among the relatively well-studied ISVs. ISOFs circulate in natural mosquito populations, and mosquitoes can also be experimentally infected using an infectious blood meal or through intrathoracic injections [10, 12, 16, 17]. Interestingly, available evidence indicates that ISOFs can modulate the propagation of dual-host (mosquitoes and vertebrates) orthoflaviviruses in mosquitoes [16–25].

VT of arboviruses occurs mainly by two mechanisms: transovarial transmission (TOT), where the virus infects the germinal tissues of the female vector, and transovum transmission, which occurs at the time of fertilization or due to viral contamination of eggs during oviposition [26]. High VTRs are believed to be due to TOT, which results from infection of developing oocytes. Once the virus establishes infection of the germinal tissue, a high proportion of progeny in successive generations are infected transovarially (defined as a stabilized infection). VT varies between virus families and even among viruses within the same genus [4, 27–35]. Orthobunyaviruses, such as La Crosse virus and San Angelo virus, display high VTRs, whereas orthoflaviviruses, such as DENV and ZIKV, and alphaviruses (e.g., CHIKV) exhibit low VTRs.

ISOFs have higher VTRs than dual-host orthoflaviviruses, indicating evolution of VT within the same genus [11, 36, 37]. Stabilized infections have been demonstrated for both orthobunyaviruses and ISOFs [32, 36, 37]. Oocyte infection has been shown for orthobunyaviruses [38, 39], but such evidence for dual-host orthoflaviviruses is rare. Previous studies failed to demonstrate the presence of orthoflaviviruses in ovarian follicles, while orthoflaviviral antigens could be easily detected in ovariole sheath cells and oviducts [40–43]. Only one study demonstrated the presence of DENV in a few oocytes in *Ae. aegypti* [6].

Cell fusing agent virus (CFAV), aptly named for its ability to fuse *Aedes albopictus* cells, was the first described ISOF infecting an *Ae. aegypti* cell line [44]. Some natural populations of *Ae. aegypti* are persistently infected with CFAV, while others do not carry the virus [10]. Field-collected mosquitoes carrying CFAV vertically transmit the virus to a large number of progeny [11, 36]. Similar high filial infection rates (FIRs) of CFAV were also observed in laboratory infected *Ae. aegypti* collected in Bangkok, Thailand [36]. Efficient VT requires TOT of the virus, where the virus infects the ovarian follicles and in turn infects the developing progeny. It is not clear how ISVs establish stabilized infections. To better understand why ISOFs and dual-host orthoflaviviruses exhibit different VTRs, we monitored VT and ovary infection patterns of CFAV and ZIKV in artificially infected laboratory-colonized *Ae. aegypti*. Our results indicated that vertical transmission of CFAV is rare in laboratory-infected mosquitoes.

## Methods

### Mosquitoes

Mexican *Ae. aegypti* (kindly provided by GD Ebel, Colorado State University) were originally collected in Poza Rica, Mexico, in 2016. Florida *Ae. aegypti* were collected in Miami-Dade County, Florida in October 2017 (kindly provided by M DeGennaro, Florida International University) and reared in the laboratory. Bangkok *Ae. aegypti* mosquitoes were obtained from Bangkok, Thailand in 2011 (kindly provided by Nikos Vasilakis, University of Texas Medical Branch, Galveston, Texas). Mosquitoes were reared in the insectary and maintained at 28 °C with 16/8-h photo/dark period. Mosquito larvae were fed ground fish food, and the adults were maintained with a 10% sucrose solution.

### Virus

ZIKV strain PRVABC59 (kindly provided by the Centers for Disease Control and Prevention, Fort Collins, CO, USA; GenBank accession no. KU501215), was initially obtained from the serum of a patient who had traveled to Puerto Rico in 2015. It was passaged three times on Vero cell culture and twice on C6/36 cell culture. CFAV (Galveston) was obtained from the World Reference Center for Emerging Viruses and Arboviruses, University of Texas Medical Branch, Galveston, TX, USA. All stock viruses were generated in C6/36 cells (American type culture collection (ATCC), CRL-1660). C6/36 cells were grown in MEM supplemented with 10% fetal bovine serum (FBS), 1.5 g/L sodium bicarbonate, 0.1 mM non-essential amino acids, and maintained at 28 °C in 5% CO_2_. All media had 100 U penicillin ml^−1^ and 100 μg streptomycin ml^−1^. Confluent monolayers of C6/36 cells in six-well plates were infected with ZIKV at 0.1 multiplicity of infection (MOI). After 1 h of adsorption, 3 ml of maintenance medium (growth medium with 2% FBS) were added to each well and the plates were incubated at 28 °C. Supernatants from each well were collected after 5 days and pooled. Freshly prepared viruses were used for all mosquito infections. CFAV infected cells were incubated at 28 °C for 7 days before harvesting.

### Mosquito infection

**Fig. 1:**
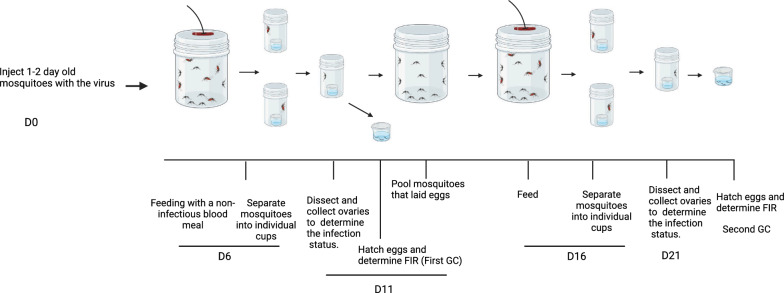
A schematic of the workflow for the vertical transmission assay during two gonotrophic cycles (GCs). This figure was created using BioRender.com

In total, 120–200, 1–2-day old *Ae. aegypti* females were injected with 100 nl of the virus suspension containing about 1000 virus RNA copy equivalents using a nanoject III microinjector. The viral RNA concentration was determined from a standard curve, which was prepared using a series of tenfold dilutions of a chemically synthesized target RNA. Each standard concentration was performed in triplicate. The stock virus was diluted with phosphate buffered saline (PBS). Injected mosquitoes were kept in a cardboard container and maintained with 10% sucrose at 28 °C with a 16/8-h photo/dark period, and 6 days post injection, mosquitoes were fed with a non-infectious sheep blood meal (Fig. 1). Engorged mosquitoes were individually separated into coffee cups with mesh on the top. Each cup contained a 30 ml plastic cup half filled with water and the remaining half had a coffee filter paper touching the water. Before feeding, 15–20 mosquitoes were collected to determine the infection rate and the spread of the virus to various tissues. The mosquitoes were dissected to determine the ovary and midgut infection patterns, and bodies were collected in 500 μl of mosquito diluent (1× PBS containing 20% heat inactivated FBS, 100 U/ml of penicillin, 100 μg/ml streptomycin, 10 μg/ml gentamicin and 1 μg/ml of Fungizone) to determine the infection status of the mosquito, and 5 days post-feeding, eggs and larvae were collected, allowed to hatch, reared to the third or fourth instar stage, and finally pooled to detect the presence of the virus among the progeny (Fig. 1).

To determine the VT in the second GC, mosquitoes that had laid eggs in the first GC were transferred to a new cardboard container and maintained with 10% sucrose at 28 °C. On day 10 post-first blood meal, they were fed again with a non-infectious sheep blood meal; engorged mosquitoes were separated into individual cups as before, and 5–6 days post feeding, eggs/larvae were collected and reared to the third/fourth instar stage, when they were pooled to determine the transmission of the virus to the progeny. Mosquitoes were dissected, ovaries and midguts were collected to determine the infection pattern by in situ hybridization (ISH) described below, and the bodies were collected to determine the infection status of the insect (Fig. 1).

To determine VT rates, 20% of the larvae derived from each mosquito were collected in 500 μl of mosquito diluent. RNA was then prepared and tested for the presence of the virus using the virus-specific primer/probe combination in a reverse transcription-quantitative polymerase chain reaction assay (RT-qPCR). The rationale behind this was that, if any pool turned out positive, then the remaining larvae would be reared to the adult stage. Adults would then be used for determining the infection status, ovary infection pattern, and transmission of the virus to subsequent generations. When none of the pools were positive, the remaining larvae from all mosquitoes were pooled in 800 μl mosquito diluent, with 15–20 larvae per pool. The presence of the virus in each pool was determined via RT-qPCR using the virus-specific primer/probe combination. VT rate (VTR) is defined as the number of infected females in a population that produce at least one infected offspring. The minimum filial infection rate (MFIR) is the total number of positive pools divided by total number of larvae. ZIKV primers: Forward, 5’ CCGCTGCCCAACACAAG-3’; Reverse, 5-CCACTAACGTTCTTTTGCAGACAT-3’; Probe, 5’Cy5/AGCCTACCTTGCCAAGCAGTCAGACACTCAA.

CFAV primers: Forward, 5’-CCATTGCGACAGAGGATTCA-3’; Reverse, 5’-GTGTCGCTAACAGAGTGGAAG-3’; Probe, 5’-/Cy5/TTCCATCGCTAGGTCAGCCATTGT-3’. S7 Primers: Forward, 5’ -ACCGCCGTCTACGATGCCA-3’; Reverse, 5’-ATGGTGGTCTGCTGGTTCTT-3’. The primers were synthesized at Integrated DNA Technologies, Coralville, IA.

### RNA isolation

Mosquito RNA (total RNA) was isolated using the *mir*Vana miRNA isolation kit (Invitrogen). cDNA synthesis and PCR amplification was carried out using the Superscript IV one-step reverse transcription polymerase chain reaction (RT-PCR) kit from Invitrogen. The PCR products were visualized by 1% agarose gel electrophoresis.

### In situ hybridization (ISH)

Following dissection, individual midguts and ovaries were fixed with 1 ml of 10% phosphate-buffered formalin in a 24-well plate and stored at 4°C for 3–7 days before processing for ISH. Both CFAV- and ZIKV-specific probes were purchased from Advanced Cell Diagnostics, Inc., 7707 Gateway Blvd., Newark, CA, 94560, and ISH was carried out following the manufacturer’s protocol. Briefly, samples derived from infected mosquitoes were pooled (6–8 tissues per pool) and then transferred to mesh inserts (Ted Pella, Prod. No. 36173) in a 12-well plate, where they were then washed with 3.0 ml 1× PBS with 0.1% Tween 20 (PBST) for 10 min. All subsequent washings were performed at room temperature (RT) on an orbital shaker for 10 min, unless otherwise specified. Tissues were then washed sequentially with 25% MeOH in PBST, 50% MeOH in PBST, 75% MeOH in PBST, and 100% MeOH. Samples were transferred to a fresh well containing 0.2 M HCl in 100% MeOH and incubated for 30 min at RT. The tissues were then washed sequentially with 75% MeOH in PBST, 50% MeOH in PBST, 25% MeOH in PBST, and PBST + 1% bovine serum albumin (BSA). Following the wash in PBST + 1% BSA, the mesh inserts were transferred to 50 ml beakers containing preheated target retrieval solutions and steamed for 15 min. The inserts were then immediately placed in PBST + 1% BSA in a 12-well plate for 1 min at RT. Immediately after, the inserts were removed and placed in 100% MeOH for 1 min at RT. The samples were then carefully removed from the mesh inserts, transferred to 1.5 ml centrifuge tubes, and washed with 1 mL of PBST + 1% BSA at RT. Next, the tissues were treated with Protease Plus in a 40°C water bath for 40 min, followed by treatment with probe diluent. After removal of the probe diluent, two drops (enough to cover the samples) of the appropriate probe was added to each tube, and the tubes were then incubated at 40°C for 2 h. The samples were then washed twice with 1 ml 1× Wash Buffer for 2 min at RT with gentle shaking. Following the washes, the samples were placed in 1 mL of 5× saline sodium citrate (SSC) and stored at RT overnight.

On the next day, the samples were washed twice with Wash Buffer at RT. Then they were sequentially treated with enough drops of AMP1, AMP2, and AMP3 to cover the tissues in each tube, and incubated in a 40°C water bath for 30 min, but 15 min for AMP3. The samples were washed with Wash Buffer at RT between treatments. After AMP3, enough drops of HRP-C1 were added to entirely cover each sample. After 15 min at 40°C, samples were washed thrice with 1× Wash Buffer at RT. Then, 200 μL of 1:1500 diluted fluorophore was added to each tube, and tubes were floated in the water bath for 30 min. Following a wash with Wash Buffer, the solution was immediately replaced with 2–3 drops of HRP blocker and incubated in a 40°C water bath for 15 min. During the last 5 min, 1 μL of 4′,6-diamidino-2-phenylindole (DAPI) (0.5 μg/ml) was added to each sample. Then the samples were washed with Wash Buffer and carefully transferred to slides (five tissues per slide), mounted with ProLong Gold Antifade Mounting medium (Invitrogen), and covered with coverslips. Slides were then dried in the dark at RT for 2 h and then stored at 4 °C or viewed microscopically. The samples were viewed and pictured at 10× magnification to check the tissue infection pattern and at 40× for follicle infection.

## Results

### Midgut and ovary infection patterns of CFAV in *Ae. aegypti* after intrathoracic (IT) inoculation

**Fig. 2:**
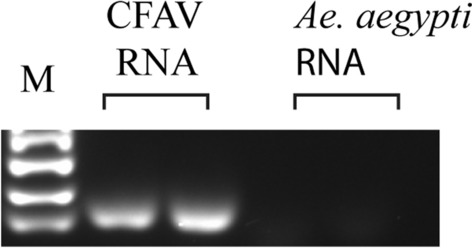
CFAV is absent in *Ae. aegypti* (MX) mosquitoes. RT-PCR was carried out using CFAV-specific primers. M, size markers; CFAV RNA in duplicate was used as a positive control. *Ae. aegypti* RNA was isolated from pools of five mosquitoes. Two separate pools were used

**Fig. 3:**
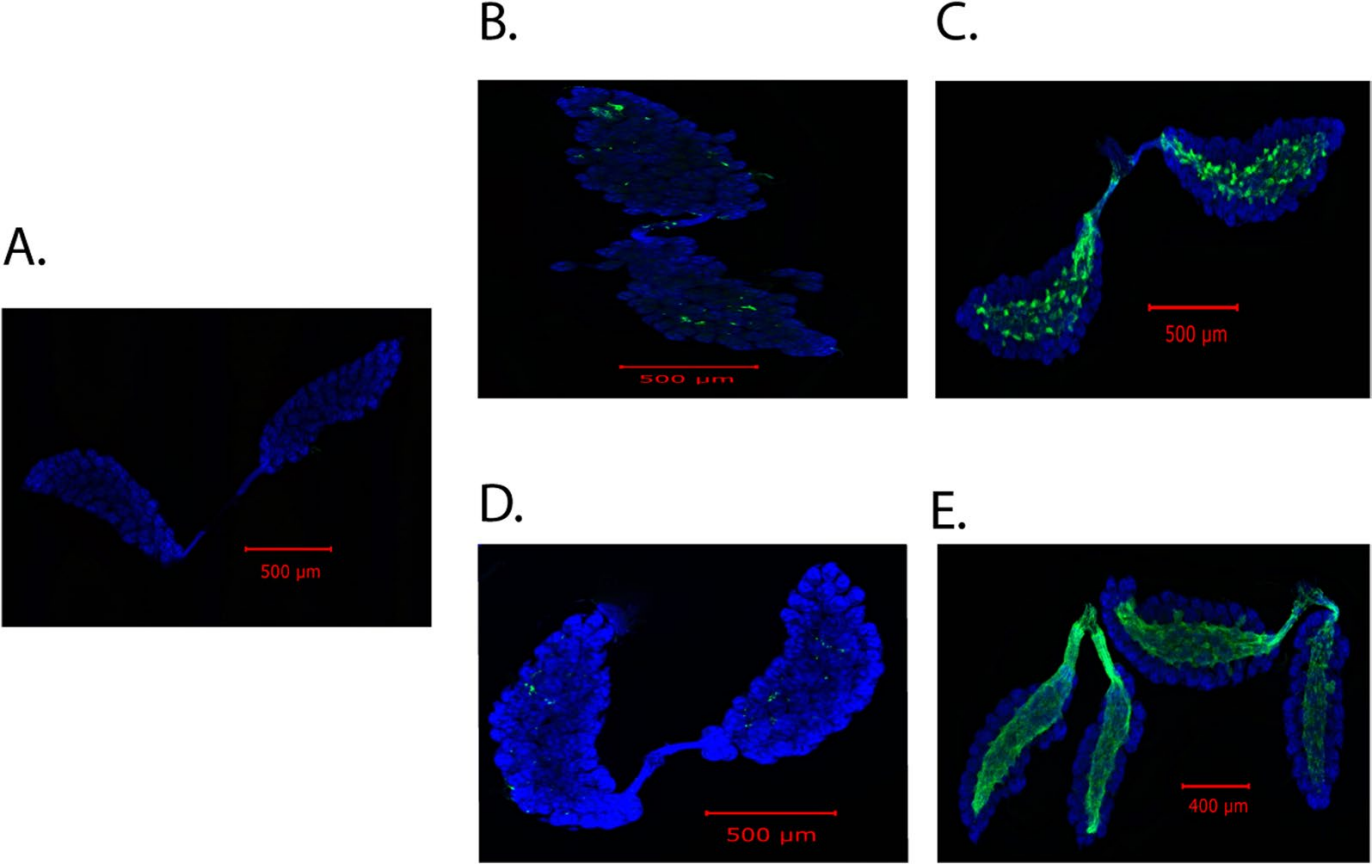
Both CFAV and ZIKV infect mosquito ovaries following IT injection. For pre-blood meal ovaries, mosquitoes were dissected 6 days post-IT injection, ovaries were fixed with 10% formalin, and the presence of the virus was visualized by ISH, using virus-specific probes. For post-blood meal ovaries, mosquitoes were dissected 5 days post-blood meal, and the presence of the virus was visualized by ISH. **A** Control uninfected ovaries; **B** and **C** CFAV-infected ovaries before and after blood meal, respectively; **D** and **E** ZIKV-infected ovaries before and after blood meal, respectively. Images were taken at 10× magnification

**Fig. 4:**
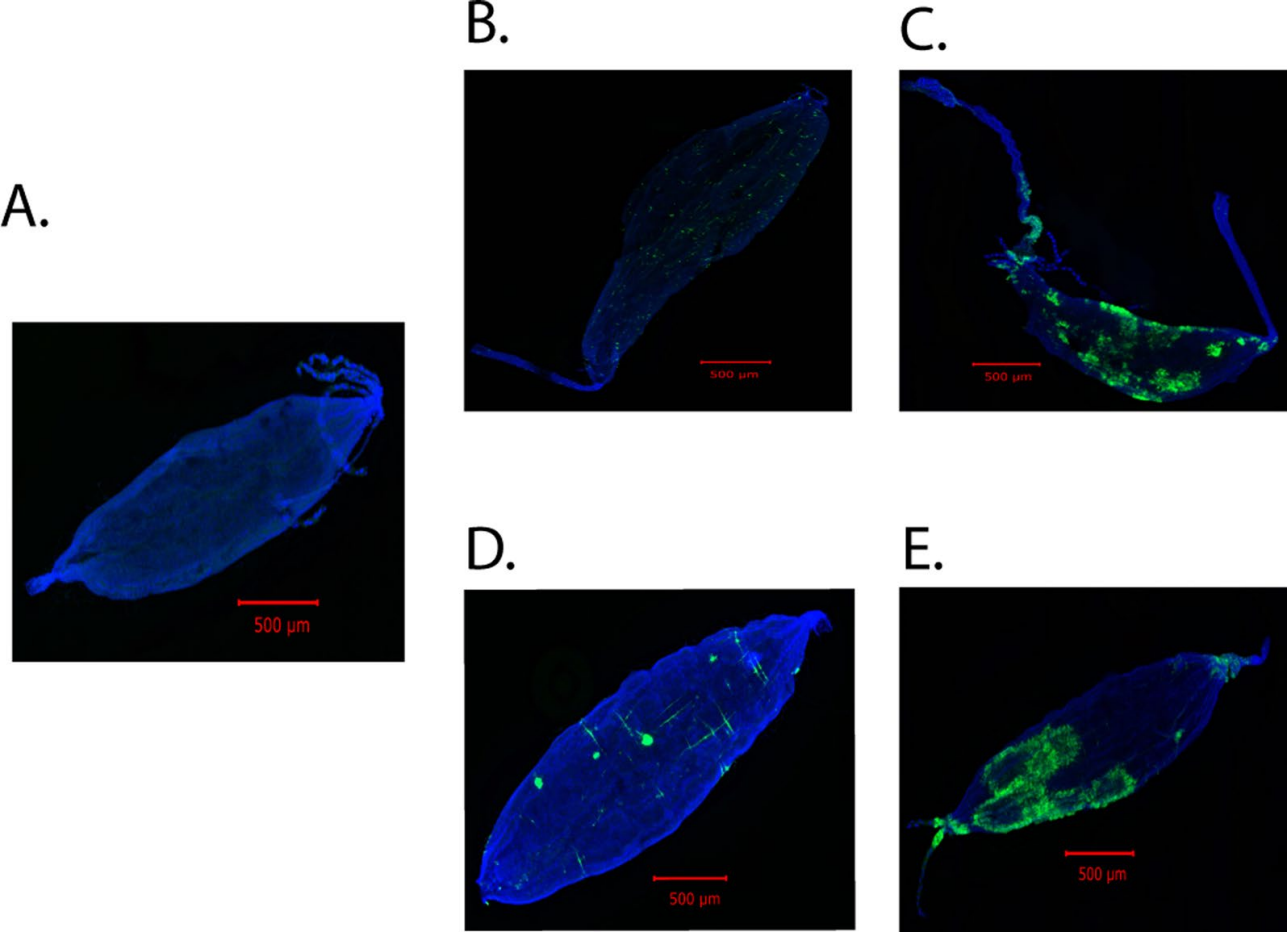
Infection of mosquito midguts by CFAV and ZIKV. Midguts were dissected as described for ovaries in Fig. 3 and visualized by ISH. **A** Control uninfected; **B** and **C** CFAV-infected midguts before and after feeding, respectively; **D** and **E** ZIKV-infected midguts before and after feeding, respectively. Images were taken at 10× magnification

The laboratory colony of *Ae. aegypti* (originated from Mexico) was first tested for the presence of CFAV to make sure that they were CFAV-free. Two pools (each containing five mosquitoes) were tested by routine RT-PCR analysis. As shown in Fig. 2, no RT-PCR product was obtained using RNA from the mosquito pools, indicating that our laboratory strain is CFAV free. To determine the midgut and ovary infection patterns, 120 *Ae. aegypti* females were injected with 1000 RNA copy equivalents of CFAV (Galveston) and then incubated at 28 °C, and 6 days post-injection, 15 mosquitoes were collected to determine the infection rate (bodies) and spread of the virus to various tissues (midguts and ovaries). In total, 15/15 (100%) mosquitoes were infected. Infection of the midguts and ovaries was determined by ISH using the CFAV-specific nucleic acid probe as described in Materials and Methods. Both the midguts and ovaries of all mosquitoes tested were infected with the virus (Figs. 3B and 4B). However, the infections in both tissues were sporadic and not widespread. In total, 11 ovary pairs were tested.

Previous studies have demonstrated that ovaries become more permeable to viral infection after a second non-infectious blood meal following an infectious blood meal [6, 43]. This is likely due to the altered morphology of the ovary due to egg formation, and the eggs formed after the first infectious blood meal are already laid before the virus reaches the reproductive tissue. On the basis of this observation, the remaining CFAV-injected mosquitoes were fed with a non-infectious blood meal 6 days post-IT inoculation. The engorged mosquitoes were separated into individual cups containing an oviposition substrate, and 5 days post-feeding, mosquitoes were dissected and the spread of infection was monitored by ISH. As before, all mosquitoes were infected with the virus; infections were extensive in both midguts and ovaries (Figs. 3C, 4C). These results suggest that CFAV infects mosquito ovaries following IT inoculation.

### ZIKV shows similar infection patterns when infected via IT inoculation

To determine whether ZIKV shows a similar infection pattern when infected via IT inoculation, we infected 125 mosquitoes with 1000 RNA copy equivalents of ZIKV, and 6 days post-infection, we tested 15 mosquitoes to determine the infection rate, along with midgut and ovary infection patterns. The remaining mosquitoes were fed with a non-infectious blood meal, and 5 days post-feeding, mosquitoes were dissected to determine the infection status. As observed with CFAV, infections of both midguts and ovaries were sporadic before feeding, but extensive after the blood meal (Figs. 3D and E, Fig. 4D and E), and 100% of mosquitoes were infected. A total of 13 post-feeding ovary pairs were tested by ISH. We also checked the infected ovaries from post-feed mosquitoes at higher magnification to determine if any of the primary follicles were infected. As shown in the supplementary Fig. 1, none of the follicles were infected.

### Vertical transmission of CFAV and ZIKV in artificially infected *Ae. aegypti*

We monitored VT of both ZIKV and CFAV in artificially infected *Ae. aegypti* (Fig. 1), and 6 days post-injection, mosquitoes were fed with a non-infectious blood meal (see above). Engorged mosquitoes were housed in individual cups containing oviposition substrates. Larvae derived from individual mosquitoes were allowed to grow until the third or fourth instar stage, when they were then tested for the presence of the virus by RT-qPCR using virus-specific primer–probe combinations. Our objective was to gather 20% of larvae derived from each mosquito in pools to test for infections. If any pool was found to be infected, the remaining larvae of that pool were raised to adulthood and the transmission of the virus was then monitored in subsequent generations.

For ZIKV, from 30 mosquitoes, none of the pools containing 20% of the larvae were infected. The remaining larvae from all the mosquitoes were pooled. There were 57 pools, each containing 15 larvae. One pool was ZIKV positive with a C_T_ value of 22.2 (Table 1), indicating that one or more progeny in that pool were infected with ZIKV. These results suggest that infected larvae were not included in the initial 20% pool and that not all progeny produced by an infected mosquito become infected with the virus. We estimate a minimum FIR (MFIR) of 1:985 [number of infected pools:total number of larvae tested, where number of larvae tested consisted of larvae in the pools (855) + initial 20% of total larvae (130)].

**Table 1:** Vertical transmission of Zika virus (ZIKV) and cell fusing agent virus (CFAV)

Virus	Experiment no.*	Gonotrophic cycle	Number of mosquitoes	Number of pools	Number of larvae/pool	Number of pools infected (C_T_ value)	MFIR^†^
ZIKV	1	1	30	57	15	1 (22.2)	1:985
CFAV	1	1	48	107	15	0	–
	2	1	82	82**		0	–
		2	36	82	20	1 (27.4)	1:1997
	3	1	47	116	18	0	–
		2	45	86	18	1 (24.6)	1:1938

*Experiments 1 and 2 were performed with *Ae. aegypti* (MX) population and experiment 3 with *Ae. aegypti* (FL) population. 100% infection in all experiments.

**Only 20% of each mosquito progeny tested

^**†**^The minimum filial infection rate (MFIR) was calculated as the number of positive pools:total number of larvae tested (larvae in pools + the initial 20% larvae tested separately)

For CFAV, none of the pools tested with 20% of the progeny were infected with the virus. As for ZIKV, the remaining larvae were pooled and tested for the presence of the virus. There were 107 pools, with 15 larvae per pool, derived from 48 mosquitoes. None of the pools were CFAV positive (Table 1). In the next experiment, we repeated this procedure for two GCs. In the first GC, initial examination of 20% of larvae (derived from 82 infected mosquitoes) turned out to be CFAV negative. A total of 44 mosquitoes that had laid eggs were then fed again with a non-infectious blood meal and tested for the presence of the virus in the progeny larvae. We tested again 20% of larvae (total 357 from all mosquitoes), and none were positive. The remaining larvae from 36 mosquitoes were pooled and tested for the presence of the viral RNA. We tested 82 pools, each pool containing 20 larvae. Only one pool turned out to be positive with a C_T_ value of 27.4 (Table 1). These results suggest CFAV rarely transmits vertically in artificially infected *Ae. aegypti*, and not all progeny produced by an infected parent are infected with the virus. The MFIR is 1:1997.

### *Ae. aegypti* Bangkok strain is CFAV positive, and rare VT transmission of CFAV is population independent

**Fig. 5:**
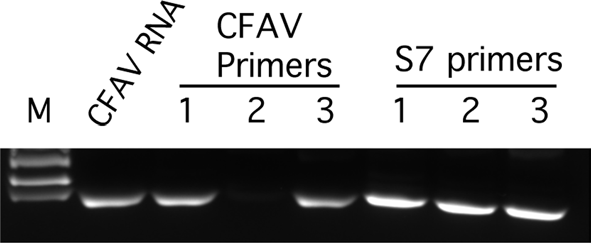
*Ae. aegypti* mosquitoes from Bangkok are infected with CFAV. Routine RT-PCR analysis was carried out with three different pools of both males and females. M, size marker; CFAV RNA was used as a positive control; 1, 2, and 3 are three mosquito pools. Lane 2 was derived from a pool containing both infected and uninfected mosquitoes. The ribosomal S7 protein-coding gene primers was used as an endogenous reference to make sure that the faint band in pool 2 was not due to any technical problems related to the sample

It is possible that VT of CFAV is mosquito population specific. Contreras-Gutierrez et al. clearly demonstrated high FIRs of CFAV in *Ae. aegypti* originated from Bangkok, Thailand [36]. Since we used the same CFAV strain in our experiments that has previously been used for the Bangkok strain [36] and we failed to demonstrate a high FIR in our strain background, we wished to repeat the same experiment in the *Ae. aegypti* Bangkok strain (which was kindly provided by Nikos Vasilakis). First, we tested these mosquitoes by routine RT-PCR to make sure that the strain was CFAV free. We tested three pools, each containing 4–5 adult mosquitoes (including both males and females). Our results, unfortunately, indicated that these mosquitoes were already infected with CFAV (Fig. 5). One pool had a faint band, suggesting that not all mosquitoes in the population were infected with the virus. We also tested 15 males and 15 females by RT-qPCR, and 3 out of 15 males and 6 out of 15 females turned out to be CFAV positive with two different primer–probe combinations.

We also have a second laboratory colony of *Ae. aegypti* collected from Florida, USA. A total of 30 mosquitoes were tested by RT-qPCR to determine whether these mosquitoes harbored CFAV. None of the tested mosquitoes were CFAV positive. We examined VT of CFAV in two GCs in the Florida strain. In the first GC, the initial 20% larvae pools derived from 47 mosquitoes were CFAV negative. The remaining larvae were collected in 116 pools, each containing 18 larvae. None of the pools were positive for CFAV. In the second GC, as before, initial pools with 20% of the larvae were CFAV negative. The remaining larvae were collected in 86 pools, with 18 larvae per pool. Only one pool turned out to be CFAV positive with a C_T_ value of 24.6 (Table 1), resulting in a MFIR of 1:1938. This result is very similar to that obtained with the Mexican population. All these results suggest that CFAV rarely transmits vertically in laboratory colonized mosquitoes.

## Discussion

Most ISVs are associated with mosquitoes, which transmit several arboviruses of public health significance. ISOFs received significant attention following the observation that the presence of ISOFs modulates the infection and transmission of several clinically important arboviruses, such as Zika, dengue, and West Nile viruses [16–25]. It is thus possible to introduce an ISOF into a mosquito vector and alter the vector’s ability to transmit various pathogenic orthoflaviviruses. To use ISOFs as a biocontrol agent, it is necessary to understand how ISOF infections are stabilized and maintained in vector mosquitoes. These ISVs can be detected in all life stages as well as in adults of both sexes in mosquitoes, suggesting that VT is the predominant mechanism of maintenance of these viruses in nature [10]. However, venereal transmission and other possible horizontal modes of transmission (e.g., transmission during the larval stages) have also been demonstrated for these viruses [11–15]. ISOFs show high VTRs in field-collected mosquitoes, whereas dual-host orthoflaviviruses exhibit low VTRs. High VTRs require viruses to infect the reproductive tissues. Accordingly, insect-specific viral RNA has been detected in the ovaries of field-collected mosquito progeny, as well as in laboratory-infected mosquito ovaries [17, 37, 45, 46]. Dual-host orthoflaviviral RNA and the presence of the viral antigen have been demonstrated in *Ae. aegypti* ovaries [6, 40–43], but VT remains low. To understand why ISOFs and dual-host orthoflaviviruses exhibit different VT rates, we monitored ovary infection pattern and VT in artificially infected *Ae. aegypti* mosquitoes.

Our results showed that CFAV, like ZIKV, infects our laboratory colony of *Ae. aegypti* following IT injection. Sporadic infections of midguts and ovaries were observed in 100% of mosquitoes tested 6 days post-IT inoculation. Infections became widespread after a non-infectious blood meal, indicating that ovaries become more permeable to infections following a blood meal (Fig. 3). As suggested previously, this was likely due to morphological alterations following egg formation [6, 43]. When mosquitoes are infected with ZIKV with an infectious blood meal, about 60% of mosquitoes are infected even after a second non-infectious blood meal [43]. Here, we observed 100% infections after IT injection. This difference is likely due to the sensitivity of the assay. Ovary infection following per os infection was monitored by indirect immunofluorescence assay, while infection after IT inoculation was analyzed by ISH. For both ZIKV and CFAV, infection of ovarian follicles was not detected among the ovaries examined (Additional file 1: Fig. S1). It is possible that infection of ovarian follicles is a rare event, and we have not examined enough ovaries to see that.

FIRs of ZIKV were very similar to that of our previous observation. Only 1 pool out of 57 pools was infected with the virus in the first GC, yielding a MFIR of 1:985 (Table 1). If all larvae in the infected pool were derived from one infected mosquito, that would result in a VTR of 3.33%, which is similar to our previous observation [43]. Following per os infection, VT of ZIKV is observed only after a second non-infectious blood meal. It is most likely due to delayed dissemination of the virus, as it takes about 7–10 days for the virus to reach the reproductive tissue and by then the eggs are already laid after the first infectious blood meal [6, 43]. In the second GC, the virus has had ample time to disseminate to the ovaries; since ovaries become more permeable to viral infection after egg formation, extensive infection of ovaries results in an increasing number of progeny being infected with the virus. In our experiments, mosquitoes were infected via IT inoculation, and as a result, the viruses had already spread to the ovaries before the first blood meal. Consequently, the first blood feeding is equivalent to the second GC of a per os infection.

CFAV, on the contrary, transmits rarely vertically. We have examined VT of CFAV in two different *Ae. aegypti* populations. CFAV was not transmitted vertically in the first GC in either population. Although the ovaries were infected with the virus, no VT of CFAV was observed in the first GC. Even in the second GC, only one pool was infected in each of the population studies, yielding a minimum FIR of 1:1997 and 1:1938, respectively. However, we cannot eliminate the possibility that the observed VT in the second GC could be attributed to the virus having had more time to replicate and spread, rather than solely being caused by the ingestion of a second blood meal. Nevertheless, the results presented here suggest that VT is rare in laboratory infected *Ae. aegypti* mosquitoes. A recent study on VT with a different strain of CFAV also came to similar conclusions with two different *Ae. aegypti* populations [46]. Rare VT was not due to the presence of endogenous viral elements (EVEs), since Zhou et al. observed that VT of CFAV is unaffected by the presence of EVEs [46].

Except results published in two studies with laboratory colonized *Ae. aegypti*, all ISVs fail to transmit vertically in laboratory colonized infected mosquitoes. In one study with Kamiti-River-virus-infected *Ae. aegypti*, a filial infection rate of 3.9% was observed among the offspring collected from the combined second and third GCs [12]. Here, the mosquitoes were infected with an infectious blood meal. It is not clear whether the infected progeny were produced in the second or the third GC. If the infected progeny were produced in the third GC, then this would be similar to our results, where infected progeny were detected only in the second GC, which is similar to the third GC in oral infections. It should be noted that there is not any evidence indicating that *Ae. aegypti* serves as a natural host of the Kamiti River virus. It was isolated from field-collected *Aedes macintoshi*. In the second study, Contreras-Gutierrez *et al*. investigated VT of CFAV in *Ae. aegypti*, collected in Bangkok, Thailand. They observed a high of FIR (30%) among the F1 mosquitoes. Unfortunately, as shown above, this strain is naturally infected with CFAV. Regrettably, Bolling et al. did not detect CFAV in the Bangkok strain for reasons unspecified [47]. Zakrzewski et al. used RNA-seq to characterize RNA metaviromes in wild-caught *Ae. aegypti* from Bangkok, Thailand [48]. This study showed that their Bangkok population was infected with CFAV, suggesting that *Ae. aegypti* from the Bangkok area are likely to be naturally infected with CFAV.

One common observation in most VT studies involving ISVs is the high prevalence of VT among field-collected mosquitoes, while such occurrences are rare to non-existent among laboratory-colonized mosquitoes [36, 37, 45, 46, and this study]. For high TOTs to occur, stabilized infections need to be established in the vectors. In field-collected mosquitoes displaying high FIRs, infections are stabilized. It is possible that TOT or the establishment of stabilized infections is regulated by genetic factor(s), and that all field-collected ISV-infected mosquitoes possess this genetic factor(s). Previous studies with orthobunyaviruses also suggested the presence of genetic factor(s) that controls their TOT in mosquitoes [28, 49, 50]. Further investigations are necessary to determine how stabilized infections are established in vector mosquitoes, and ISVs provide an excellent tool for these studies. Mosquitoes with stabilized infections may provide a mechanism of viral maintenance during adverse environmental conditions.

## Conclusions

Ovary infection patterns and VT of ZIKV and CFAV were analyzed to determine why these two viruses exhibit such different FIRs. Both ZIKV and CFAV infect *Ae. aegypti* ovaries following IT inoculation. These findings support the results of previous studies reporting the presence of CFAV RNA in reproductive tissues. For both viruses, infections were sporadic initially, then became widespread following a non-infectious blood meal. Similar sporadic infections were also observed in the midgut. Ovarian follicles showed no infection by either virus among the ovaries tested by ISH. VT of ZIKV was similar to previously reported results. VT of CFAV, on the contrary, remained rare. No infected progeny were observed in the first GC. In the second GC, an average minimum FIR of about 1:2000 was noted for CFAV. Rare FIRs of CFAV are mosquito population independent, as similar results were obtained with two different populations. These results suggest that it remains unclear how stabilized infections develop in mosquitoes, highlighting the need for additional studies to elucidate the mechanism behind the observed high FIRs of arboviruses in field-collected mosquitoes.

### Supplementary Information

**Additional file 1: Fig. S1.** Absence of CFAV and ZIKV in ovarian follicles. Post-feed ovaries were visualized. Images were taken at 40× magnification. **A** Control uninfected ovary; **B** and **C** CFAV-infected ovaries; **D** and **E** ZIKV-infected ovaries.

## Data Availability

All data generated or analyzed during this study are included within the article.

## Abbreviations

ATCC: American type culture collection
BSA: Bovine serum albumin
CFAV: Cell fusing agent virus
DAPI: 4′,6-diamidino-2-phenylindole
DENV: Dengue virus
EVE: Endogenous viral elements
FBS: Fetal bovine serum
FIR: Filial infection rate
GC: Gonotrophic cycle
ISH: In situ hybridization
ISOF: Insect-specific orthoflavivirus
ISV: Insect-specific virus
IT: Intrathoracic
MFIR: Minimum filial infection rate
PBS: Phosphate buffered saline
RT: Room temperature
RT-PCR: Reverse-transcriptase polymerase chain reaction
RT-qPCR: Reverse-transcriptase quantitative polymerase-chain reaction
SSC: Saline sodium citrate
TOT: Transovarial transmission
VT: Vertical transmission
VTR: Vertical transmission rate
ZIKV: Zika virus

## References

[1] Fine PE (1975). Vectors and vertical transmission: an epidemiologic perspective. Ann NY Acad Sci.

[2] Mink GI (1993). Pollen-transmitted and seed-transmitted viruses and viroids. Ann Rev Phytopathol.

[3] Lipsitch M, Siller S, Nowak MA (1996). The evolution of virulence in pathogens with vertical and horizontal transmission. Evolution.

[4] Lequime S, Paul RE, Lambrechts L (2016). Determinants of arbovirus vertical transmission in mosquitoes. PLoS Pathog.

[5] Ciota AT, Bialosuknia SM, Ehrbar DJ, Kramer LD (2017). Vertical transmission of Zika virus by *Aedes*
*aegypti* and *Ae.*
*albopictus* mosquitoes. Emerg Infect Dis.

[6] Sánchez-Vargas I, Harrington LC, Doty JB, Black WC, Olson KE (2018). Demonstration of efficient vertical and venereal transmission of dengue virus type-2 in a genetically diverse laboratory strain of *Aedes aegypti*. PLoS Negl Trop Dis.

[7] Molina-Cruz A, Gupta L, Richardson J, Bennett K, Black W, Barillas-Mury C (2005). Effect of mosquito midgut trypsin activity on dengue-2 virus infection and dissemination in *Aedes aegypti*. Am J Trop Med Hyg.

[8] Pacidonio EC, Caragata EP, Alves DM, Marques JT, Moreira LA (2017). The impact of *Wolbachia* infection on the rate of vertical transmission of dengue virus in Brazilian *Aedes aegypti*. Parasit Vectors.

[9] Altinli M, Soms J, Ravallec M, Justy F, Bonneau M, Weill M, Gosselin-Grenet AS, Sicard M (2019). Sharing cells with Wolbachia: the transovarial vertical transmission of *Culex pipiens* densovirus. Environ Microbiol.

[10] Blitvich BJ, Firth AE (2015). Insect-specific flaviviruses: a systematic review of their discovery, host range, mode of transmission, superinfection exclusion potential and genomic organization. Viruses.

[11] Logan RAE, Quek S, Muthoni JN, von Eicken E, Brettell LE, Anderson ER, Villena MEN, Hegde S, Patterson GT, Heinz E, Hughes GL, Patterson EI (2022). Vertical and horizontal transmission of cell fusing agent virus in *Aedes aegypti*. Appl Environ Microbiol.

[12] Lutomiah JJ, Mwandawiro C, Magambo J, Sang RC (2007). Infection and vertical transmission of Kamiti river virus in laboratory bred *Aedes aegypti* mosquitoes. J Insect Sci.

[13] Bolling BG, OleaPopelka FJ, Eiden L, Moore CG, Blair CD (2012). Transmission dynamics of an insect-specific flavivirusin a naturally infected Culex pipiens laboratory colony and effects of co-infection on vector competence for West Nile virus. Virology.

[14] Ye G, Wang Y, Liu X, Dong Q, Cai Q, Yuan Z, Xia H (2020). Transmission competence of a new mesonivirus, Yichang virus, in mosquitoes and its interference with representative flaviviruses. PLoS Negl Trop Dis.

[15] Peinado SA, Aliota MT, Blitvich BJ, Bartholomay LC (2022). Biology and transmission dynamics of Aedes Flavivirus. J Med Entomol.

[16] Baidaliuk A, Miot EF, Lequime S, Moltini-Conclois I, Delaigue F, Dabo S, Dickson LB, Aubry F, Merkling SH, Cao-Lormeau V-M, Lambrechts L (2019). Cell fusing agent virus reduces arbovirus dissemination in *Aedes aegypti* mosquitoes *in vivo*. J Virol.

[17] Koh C, Henrion-Lacritick A, Frangeul L, Saleh M-C (2021). Interactions of the insect-specific palm creek virus with Zika and chikungunya viruses in *Aedes* mosquitoes. Microorganisms.

[18] Vasilakis N, Tesh RB (2015). Insect-specific viruses and their potential impact on arbovirus transmission. Curr Opin Virol.

[19] Hobson-Peters J, Yam AWY, Lu JWF, Setoh YX, May FJ, Kurucz N, Walsh S, Prow NA, Davis SS, Weir R, Melville L, Hunt N, Webb RI, Blitvich BJ, Whelan P, Hall RA (2013). A new insect-specific flavivirus from northern Australia suppresses replication of West Nile virus and Murray Valley encephalitis virus in co-infected mosquito cells. PLoS ONE.

[20] Ohlund P, Lunden H, Blomstrom AL (2019). Insect-specific virus evolution and potential effects on vector competence. Virus Genes.

[21] Goenaga S, Kenney JL, Duggal NK, Delorey M, Ebel GD, Zhang B, Levis SC, Enria DA, Brault AC (2015). Potential for co-infection of a mosquito-specific flavivirus, Nhumirim virus, to block West Nile virus transmission in mosquitoes. Viruses.

[22] Hall-Mendelin S, McLean BJ, Bielefeldt-Ohmann H, Hobson-Peters J, Hall RA, van den Hurk AF (2016). The insect-specific Palm Creek virus modulates West Nile virus infection in and transmission by Australian mosquitoes. Parasit Vectors.

[23] Zhang G, Asad S, Khromykh AA, Asgari S (2017). Cell fusing agent virus and dengue virus mutually interact in *Aedes aegypti* cell lines. Sci rep.

[24] Talavera S, Birnberg L, Nuñez AI, Muñoz-Muñoz F, Vázquez A, Busquets N (2018). Culex flavivirus infection in a *Culex pipiens* mosquito colony and its effects on vector competence for Rift Valley fever phlebovirus. Parasit Vectors.

[25] Romo H, Kenney JL, Blitvich BJ, Brault AC (2018). Restriction of Zika virus infection and transmission in *Aedes aegypti* mediated by an insect-specific flavivirus. Emerg Microbes Infect.

[26] Tesh RB, Bolling BG, Beaty BJ, Beaty BJ. Role of vertical transmission in mosquito-borne arbovirus maintenance and evolution. In arboviruses-Molecular Biology, Evolution, and Control. Vasilakis N, Gubler DJ Eds. Caister Academic Pres: Norfolk UK, 2016.

[27] Miller BR, DeFoliart GR, Yuill TM (1977). Vertical transmission of La Crosse virus (California encephalitis group): transovarial and filial infection rates in *Aedes triseriatus* (Diptera: Culicidae). J Med Entomol.

[28] Tesh RB, Shroyer DA (1980). The mechanism of arboviral transovarial transmission in mosquitoes: San Angelo virus in *Aedes albopictus*. Am J Trop Med Hyg.

[29] Grunnill M, Boots M (2016). How important is vertical transmission of dengue viruses by mosquitoes (Diptera: *Culicidae*)?. J Med Entomol.

[30] Watts DM, Harrison BA, Pantuwatana S, Klein TA, Burke DS (1985). Failure to detect natural transovarial transmission of dengue viruses by *Aedes aegypti* and *Aedes albopictus* (Diptera: Culicidae). J Med Entomol.

[31] Anderson JF, Main AJ, Cheng G, Ferrandino FJ, Fikrig E (2012). Horizontal and vertical transmission of West Nile virus Genotype NY99 by *Culex salinarius* and genotypes NY99 and WN02 by *Culex tarsalis*. Am J Trop Med Hyg.

[32] Reese SM, Mossel EC, Beaty MK, Beck ET, Geske D, Blair CD, Beaty BJ, Black WC (2010). Identification of super-infected *Aedes triseriatus* mosquitoes collected as eggs from the field and partial characterization of the infecting La Crosse viruses. Virology J.

[33] Thangamani S, Huang J, Hart CE, Guzman H, Tesh RB (2016). Vertical transmission of Zika virus in *Aedes aegypti* mosquitoes. Am J Trop Med Hyg.

[34] Comeau G, Zinna RA, Scott T, Ernst K, Walker K, Carriere Y, Riehle MA (2020). Vertical transmission of Zika virus in *Aedes aegypti* produced potentially infectious progeny. Am J Trop Med Hyg.

[35] Zimler RA, Alto BW (2023). Vertical transmission of Zika virus by Florida *Aedes*
*aegypti* and *Ae.*
*albopictus*. Insects.

[36] Contreras-Gutierrez MA, Guzman H, Thangamani S, Vasilakis N, Tesh RB (2017). Experimental infection with and maintenance of cell fusing agent virus (*Flavivrus*) in *Aedes aegypti*. Am J Trop Med Hyg.

[37] Saiyasombat R, Bolling BG, Brault AC, Bartholomay LC, Blitvich BJ (2011). Evidence of efficient transovarial transmission of *Culex* flavivirus by *Culex pipiens* (Diptera: Culicidae). J Med Entomol.

[38] Tesh RB, Cornet M (1981). The location of San Angelo virus in developing ovaries of transovarially infected *Aedes albopictus* mosquitoes as revealed by fluorescent antibody technique. Am J Trop Med Hyg.

[39] Darby CS, Featherston KM, Lin J, Franz AWE (2023). Detection of La Crosse virus in situ and individual progeny to assess the vertical transmission potential in *Aedes albopictus* and *Aedes aegypti*. Insects.

[40] Doi R (1970). Studies on the mode of development of Japanese encephalitis virus in some group of mosquitoes by the fluorescent antibody technique. Jap J Exp Med..

[41] Whitfield SG, Murphy FA, Sudia WD (1973). St. Louis encephalitis virus: an ultrastructural study of infection in a mosquito vector. Virology.

[42] Kuberski T (1979). Fluorescent antibody studies on the development of dengue 2 virus in *Aedes albopictus* (Diptera: Culicidae). J Med Entomol.

[43] Nag DK, Payne AF, Dieme C, Ciota AT, Kramer LD (2021). Zika virus infects *Aedes aegypti* ovaries. Virology.

[44] Stollar V, Thomas VL (1975). An agent in the *Aedes aegypti* cell line (Peleg) which causes fusion of *Aedes albopictus* cells. Virology.

[45] Joseph RE, Urakova N, Werling KL, Metz HC, Montanari K, Rasgon JL (2023). *Culex tarsalis* is a competent host of the insect-specific alphavirus Eilat virus (EILV). J Virol.

[46] Zhou N, Huang E, Guo X, Xiong Y, Xie J, Cai T, Du Y, Wu Q, Guo S, Han W, Zhang H, Xing D, Zhao T, Jiang Y (2023). Cell fusing agent virus isolated from Aag2 cells does not vertically transmit in aedes aegypti via artificial infection. Parasit Vectors.

[47] Bolling BG, Vasilakis N, Guzman H, Widen SG, Wood TG, Popov VL, Thangamani S, Tesh RB (2015). Insect-specific viruses detected in laboratory mosquito colonies and their potential implications for experiments evaluating arbovirus vector competence. Am J Trop Med Hyg.

[48] Zakrzewski M, Rasic G, Darbro J, Krause L, Poo YS, Filipovic I, Parry R, Asgari S, Devine G, Suhrbier A (2018). Mapping the virome in wild-caught *Aedes aegypti* from Cairns and Bangkok. Sci Rep.

[49] Shroyer DA (1986). Transovarial maintenance of San Angelo virus in sequential generations of *Aedes albopictus*. Am J Trop Med Hyg.

[50] Graham DH, Holmes JL, Higgs S, Beaty BJ, Black WC (1999). Selection of refractory and permissive strains of *Aedes triseriatus* (Diptera:Culicidae) for transovarial transmission of La Crosse virus. J Med Entomol.

