# Supplementary figures and images for: Cell fusing agent virus rarely transmits vertically in artificially infected laboratory-colonized Aedes aegypti mosquitoes
Source: Parasit Vectors. 2024 Apr 4;17:177. doi: 10.1186/s13071-024-06232-6 (PMC10996217; doi:10.1186/s13071-024-06232-6)

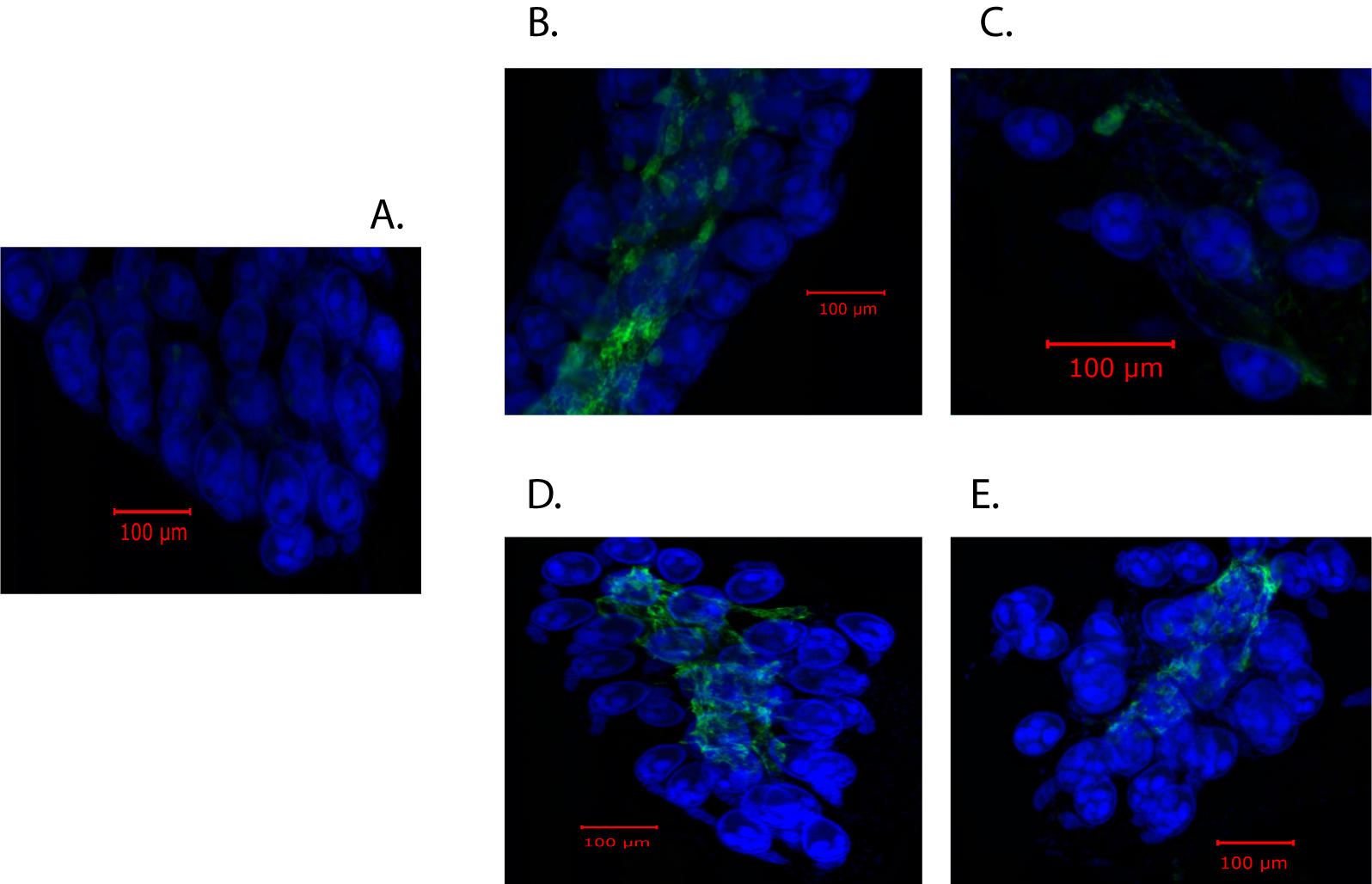

Supplement: Supplementary file 1 — Additional file 1: Fig. S1. Absence of CFAV and ZIKV in ovarian follicles. Post-feed ovaries were visualized. Images were taken at 40× magnification. A Control uninfected ovary; B and C CFAV-infected ovaries; D and E ZIKV-infected ovaries. [file 13071_2024_6232_MOESM1_ESM.tif]
